# Transoral removal of submandibular hilar lithiasis: results on the salivary duct system, glandular parenchyma, and quality-of-life recovery

**DOI:** 10.1007/s00405-023-08081-y

**Published:** 2023-07-06

**Authors:** Alvaro Sánchez Barrueco, Ignacio Alcalá Rueda, Cristina Ordoñez González, Beatriz Sobrino Guijarro, Jessica Santillán Coello, Gonzalo Díaz Tapia, Félix Guerra Gutiérrez, Alfonso Campos González, Alessandra Brenna, Carlos Cenjor Españo, José Miguel Villacampa Aubá

**Affiliations:** 1 Medicine Faculty, Alfonso X El Sabio University, Villanueva de La Cañada, Madrid, Spain; 2 ENT and Cervicofacial Surgery Department, Fundación Jiménez Díaz University Hospital and General Villalba University Hospital, Madrid, Spain; 3 Radiology Department, Fundación Jiménez Díaz University Hospital, Madrid, Spain; 4 Radiology Department, General Villalba University Hospital, Madrid, Spain

**Keywords:** Hilar, Stone, Transoral, Ductal recovery, Quality-of-life

## Abstract

**Objective(s):**

To confirm that hilar transoral submandibular sialolitectomy (TOSL) is the first treatment option for submandibular hilar lithiasis (SHL) in terms of glandular parenchyma recovery, salivary system restoration, and patient quality of life (QoL) improvement.

**Methods:**

Depending on whether the stone was easily palpable, TOSL was carried out with or without sialendoscopy. For the first time in the literature, Magnetic Resonance Sialography (MR-Si) was performed before and after TOSL, to evaluate stone characteristics, glandular parenchyma status, hilum dilation and main duct recanalization. Radiological data was examined independently by two radiologists. COSQ, a recently validated and specific questionnaire, was used to assess associated QoL.

**Results:**

Between 2017 and 2022, 29 TOSL patients were examined. With a high interobserver correlation, MR-Si was confirmed as a very useful radiological test in the pre- and post-surgical evaluation of SHL. The salivary main duct was completely recanalized in all cases. The presence of lithiasis was found in 4 patients (13.8%). After surgery, the majority of patients (79.31%) had hilum dilation. There was a statistically significant improvement in parenchyma status, but no significant progression to glandular atrophy. After surgery, COSQ mean values always improved (22.5 to 4.5).

**Conclusions:**

TOSL is the ideal surgical technique for the management of SHL, resulting in improved parenchymal inflammatory changes, recanalization of Wharton’s duct, and enhancement patients’ QoL. As a result, before removing the submandibular gland, TOSL should be considered as the first treatment option for SHL.

## Introduction

Sialolithiasis is one of the most common causes of chronic obstructive sialadenitis (COS). These calculi primarily affect the submandibular gland (about 82%) [1–3] and the specific hilar location on submandibular gland varies according to the series, from 24.4% [3] to 53% [2]. Bilateral cases are a rare condition that represents only 3% of all occurrences [4].

Submandibular hilar lithiasis (SHL) has classically been resolved by glandular excision, a direct transoral approach or, more recently, by sialendoscopy. Due to the characteristic large size of SHL [3], sialendoscopy is not usually the first choice as a single treatment option.

There have been a number of justifications for the removal of salivary gland, including the fact that COS invariably alters the glandular parenchyma's functionality and the potentially simple development of new calculi [5]. Furthermore, submandibular gland excision, which is a well-standardized technique, has no remarkable negative impact on patients' quality of life. Apart from these considerations, the transoral approach technique is sometimes avoided and underappreciated due to its theoretical technical complexity, the risk of lingual nerve injury, and the widespread usage of the submandibulectomy surgery. Yet, salivary gland removal is not without risks, such as lingual nerve damage, cutaneous scarring, and the consequent volume defect.

Despite ongoing debate, a transoral approach with or without sialendoscopy assistance is recommended to resolve symptomatic, impacted, and palpable SHL [5–13]. Additionally, it has been demonstrated to have high success rates, few side effects, and a low recurrence rate [5, 7, 8, 14].

Most authors do not propose suturing the duct opening following stone removal [5, 8, 10, 15], due to the risk of possible post-surgical stenosis; nor do they recommend performing a sialodochoplasty, which is a new salivary opening in the floor of the mouth [6]. However, others perform sialodochoplasty, or the marsupialization of the duct, with very good results [11].

Only a few series study what happens to the Wharton’s hilar duct following surgery. They used sialography to assess the structural results of the salivary system after surgery, but no information about parenchymal changes was provided [6, 8].

With this research, we pretend to confirm that the hilar transoral submandibular sialolitectomy (TOSL) is the best treatment option for SHL [5, 6, 12, 13]. To that end, precise information regarding status of glandular parenchyma, persistence of lithiasis, evaluation of duct recanalization and the impact on the patient's quality of life will be provided. For all of this, a highly sensitive and specific test for the study of salivary pathology, such as magnetic resonance with sialography protocol (MR-Si) [16], was utilized for the first time in the literature; and a recent validated and specific questionnaire was applied.

## Materials and methods

A radiological diagnostic of SHL, symptoms consistent with COS and a surgical justification for TOSL were required for inclusion. The presence of metallic artifacts that hindered proper radiological control by MR, a lack of follow-up, or the patient's reluctance to participate in the study were exclusion criteria.

**Fig. 1:**
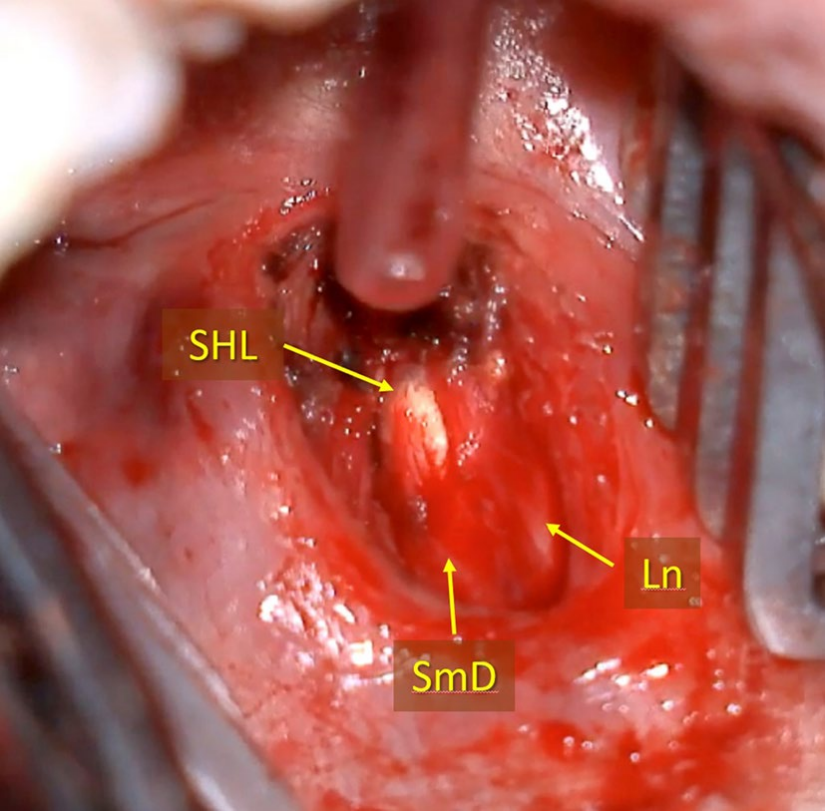
TOSL approach in left SHL. Submandibular duct (SmD) incision can be seen on the surface of the stone, and its lateral relationship with the lingual nerve (Ln)

**Fig. 2:**
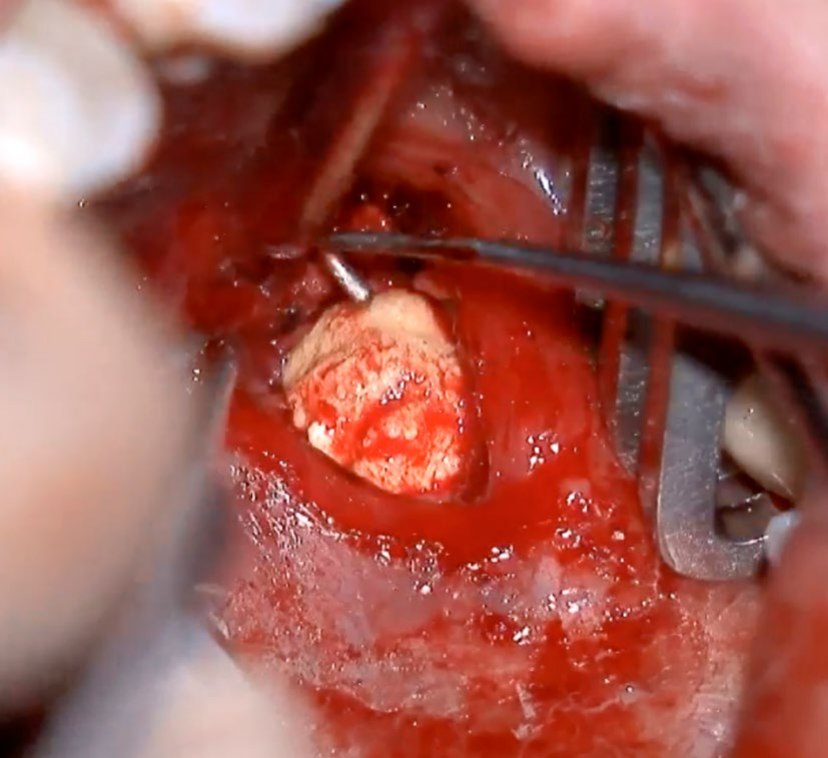
TOSL approach in left SHL. Extraction of the lithiasis with the 90-degree hook

The patients included in the study presented COS, consisting of at least two episodes of submandibular swelling; or an acute episode with an Emergency Department diagnosis of SHL.In all the cases, TOSL was conducted under general anaesthesia with nasotracheal intubation. A direct transoral approach was performed without preliminary sialendoscopy when the stone was palpable. In cases where the stone was difficult to palpate, endoscopy was employed to detect the lithiasis and the hilar region was addressed transorally using transillumination. The TOSL technique used was similar to that published by other authors [5, 9, 13]. The assistant’s collaboration is essential throughout the surgery, by drawing the tongue medially and pressing the gland externally towards the floor of the mouth. The lingual nerve is laterally detached and the duct opening is performed above the stone's surface using meticulous dissection (Fig. 1). The lithiasis is extracted using a 90-degree hook, which separates the stone from the canal walls (Fig. 2). It is critical at this stage to wash the incision, the hilum, and the canal with physiological saline solution to remove any microlithiasis or detritus. The duct was never sutured, and no stent was ever placed. Without undergoing sialodochoplasty, a single mucosal stitch was used to approximate the mucosal edges.

**Fig. 3:**
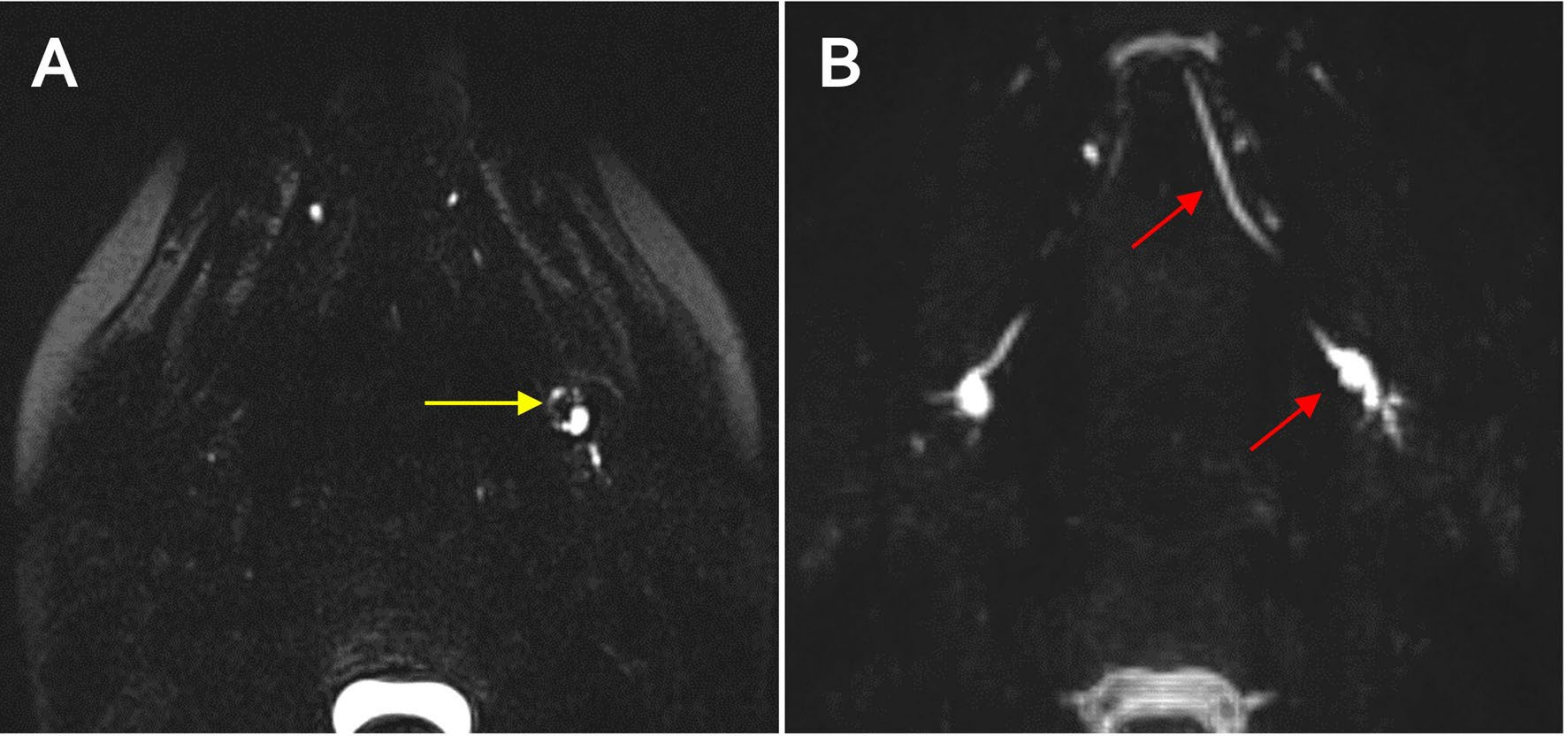
Axial STIR MR-Si. Preoperative (**A**) with left SHL (yellow arrow) and retrograde hilar dilation. Postoperative (**B**) with no SHL, low hilar residual dilation and complete recanalization of the duct (red arrows)

**Fig. 4:**
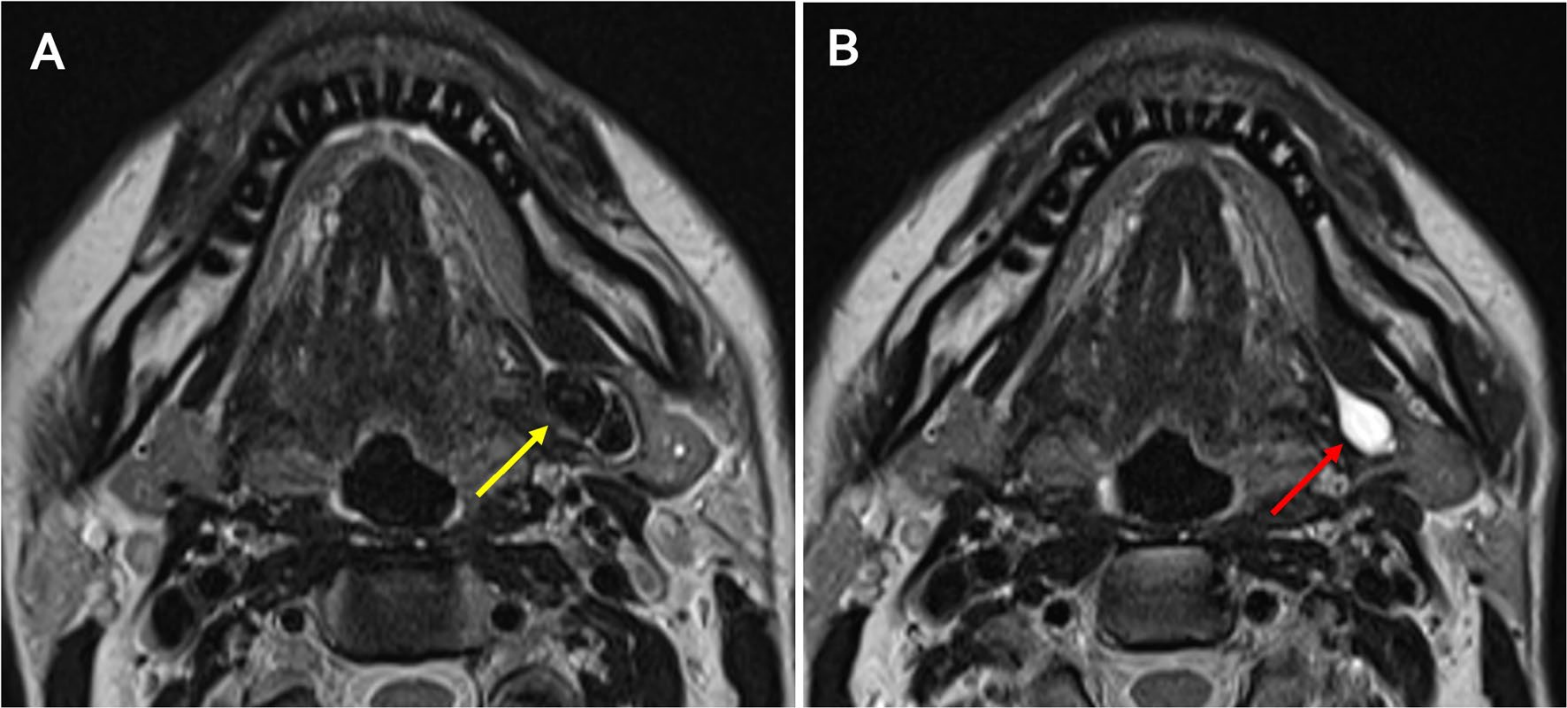
Axial T2 MR-Si with left SHL (yellow arrow). Preoperative study (**A**) with SHL with normal parenchyma and postoperative study (**B**) with absence of SHL and hilar residual dilation (red arrow)

**Fig. 5:**
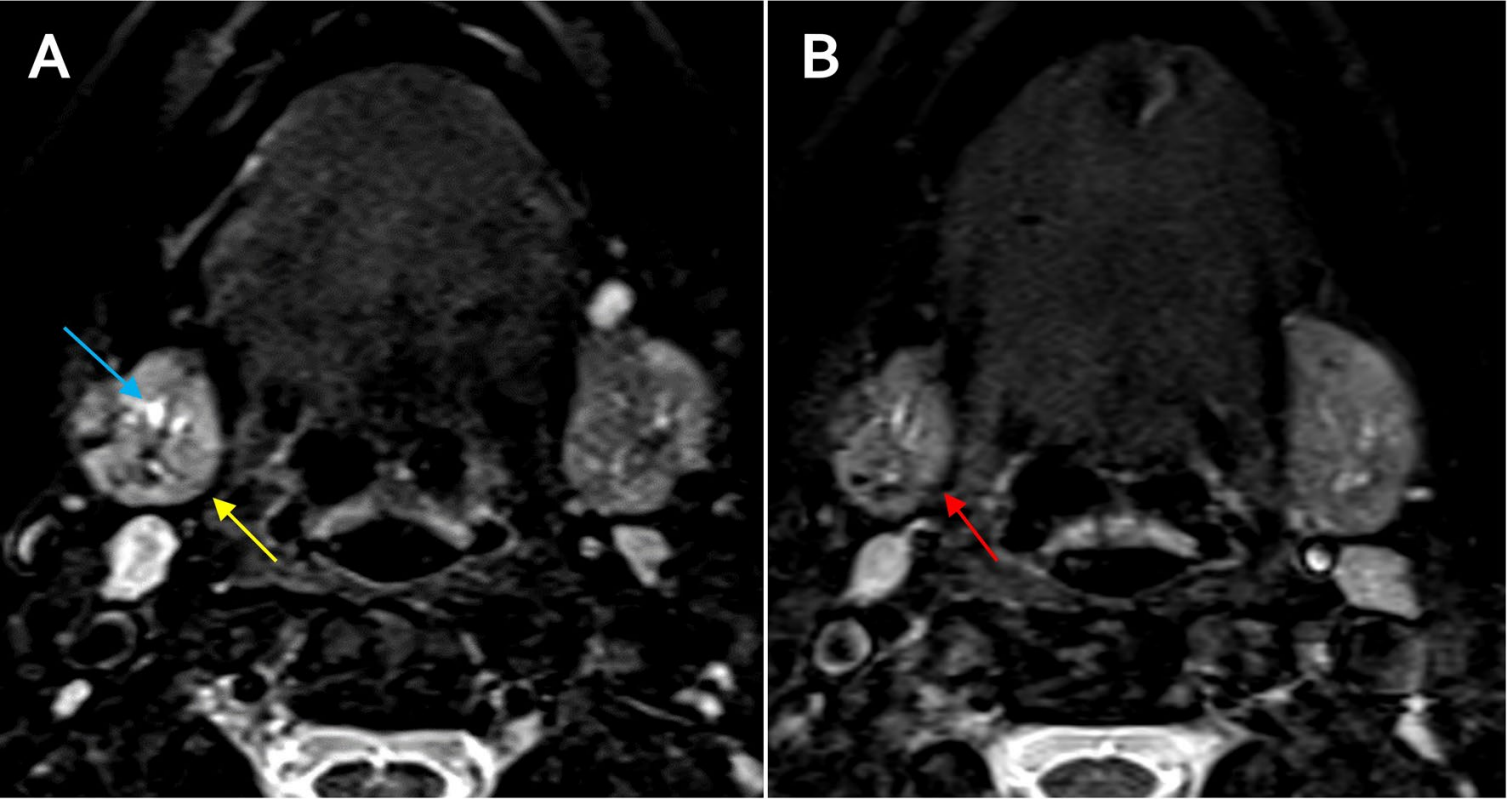
Axial STIR MR-Si. Preoperative study (**A**) with right SHL, retrograde hilar dilation (blue arrow) with parenchymal inflammatory changes (yellow arrow). Postoperative study (**B**) with resolution of inflammatory changes (red arrow)

The radiological exam was always carried out at least 45 days after an acute episode of sialadenitis. Preoperative MR-sialo was performed on all patients to confirm the hilar location of the lithiasis, and postoperative MR-sialo was performed 6 months following surgery. The MR-Si protocol adhered to the standards of the Radiology Department. MR was performed on a 3.0 T MR scanner (Siemens Magneton Verio Syngo) or a 1.5 T MR scanner (Siemens Magneton Aera). Prior to the study, the patient was given a sialagogue (half lemon juice) to stimulate salivation. Conventional MR images were obtained to assess the glandular parenchyma with spin-echo sequences as follows: axial and coronal T1-weighted (TR/TE = 500 ms/14 ms), T2-weighted (TR/TE = 2600 ms /80 ms) and short inversion time–inversion recovery (STIR) (TR/TE = 2000 ms/14 ms, TI = 160 ms). For evaluating the salivary duct system, specific MR sialography sequences were obtained: three-dimensional (3D) constructive interference in steady state (CISS) and half-Fourier acquisition single-shot turbo-spin echo (HASTE) sequences. The radiological data were examined separately by two radiologists (observer 1 and 2) with at least 10 years of experience in MR-Si. The state of the glandular parenchyma was graded as normal, undergoing inflammatory changes, or glandular atrophy. The preoperative study included hilar stone size, hilar dilation, and parenchyma status. The postoperative study evaluated the residual hilar dilatation, the persistence of any residual stone, and evidence of Wharton's duct recanalization (Figs. 3, 4 and 5).

From July 2018 onwards, the specific and validated quality of life questionnaire for chronic obstructive sialadenitis (CSOC) was implemented for preoperative and postoperative monitoring of quality of life [17]. This questionnaire, Chronic Obstructive Sialadenitis Questionnaire (COSQ) in English language, explores the different spheres of impairment in the quality of life, based on Likert-type scales. Using 18 questions and answers from 0 to 4, it covers a range from 0 (no symptoms) to 72 (maximum symptoms). Thus, from that date on, the patients who underwent surgery answered the COSQ before and 3 months after surgery.

SPSS software (v22) was used to perform statistical analysis on the sample. Because none of the variables in the sample had a normal distribution, all of the tests were nonparametric. The mean, median, mode, standard deviation, and variance for each of the quantitative measurements taken by the observers, as well as the mean of both observers’ measurements, were calculated. The Kappa index was calculated to assess the random effect of both observers' measurements. Normal, inflammatory changes, and atrophic parenchymal status were assigned ordinal values of 1, 2, and 3 to reflect the parenchymal status, respectively, which enabled the creation of contingency tables to examine the percentage of patients who improved after surgery based on each observer separately. Given the low count [5] in some of the boxes, the significance of these data was calculated using Fisher’s exact test. The correlation between several variables before and after surgery was studied using Spearman's correlation coefficient, including lithiasis size, hilum dilatation and lithiasis size, as well as the COSQ score before and after surgery and their difference.

## Results

Between March 2017 and March 2022, 36 submandibular hilar transoral sialolitectomies were performed at the Fundación Jiménez Díaz and Villalba University General Hospitals, with 29 patients fully meeting inclusion criteria. The primary exclusion criterion was the lack of a postoperative radiological control test. There were no definitive changes in tongue sensitivity or complications at the cervical level or at the base of the tongue in any of the patients.

### Epidemiological data

The mean age at the time of surgery was 45.65 years (range 19–74 years), with a homogeneous distribution by gender. The right side was operated on 72.4% of the patients (*n* = 21).

### Pre-surgical data

The presence of lithiasis was fully confirmed by both observers, with a mean size of 5.12 mm (range 1.65–10 mm), with a very good interobserver correlation (0.942).

The state of the glandular parenchyma was mostly described as normal or with inflammatory changes. The correlation was complete in cases where the glandular parenchyma was described as atrophic (*n* = 3), presenting greater interobserver variability between inflammatory and normal cases, reaching, however, a very good correlation (0.825).

The pre-surgical dilatation of the hilum was congruent with the size of the lithiasis, presenting a mean dilatation of 6.28 mm (range 2.15–10.5 mm), with a very good interobserver correlation (0.918).

### Post-surgical data

In all cases, MR-Si confirmed the recanalization of the salivary ductal system (Fig. 3), without any hilum-level obstruction. Persistence of lithiasis was evidenced in 4 patients (13.8%), all of which were noticeably smaller, less than 2 mm. In all these patients, with or without symptoms, a revision sialendoscopy was considered in order to successfully remove the residual lithiasis endoscopically at least 1 year after the TOSL. During this procedure, the closure of the previous transoral approach and the presence of fixed lithiasis adhered to the hilar mucosa were confirmed.

Most patients (79.31%) presented a hilum dilation after surgery (Fig. 4 ), with a strong interobserver agreement on this finding (0.791). In addition, this dilatation reached a mean of 3.44 mm (range 1–7.4 mm), with a very strong interobserver correlation (0.967).

The parenchymal improvement judged by each observer was subjected to a qualitative analysis, with a good interobserver agreement (0.791). Observer 1 confirmed improvement of parenchymal status from inflammatory changes to a normal status, in 56.3% of cases (*p* < 0.01). 90% of patients with normal preoperative parenchyma had statistically significant (*p* ≤ 0.01) stability for this observer. Likewise, observer 2 confirmed resolution of parenchymal inflammatory changes in 58.3% of cases (*p* < 0.01) (Fig. 5). For this observer, the stability of 58.3% of patients with normal preoperative parenchyma was also confirmed to be statistically significant (*p* ≤ 0.01). The parenchymal status worsened from inflammatory changes to atrophy was only reported in 12.5% of cases for observer 1 and 16.7% of cases for observer 2. This regression, in both cases, results statistically insignificant.

### Quality-of-life data

The COSQ was filled out completely by 22 patients (75.86%). Preoperatively the mean value of COSQ was 22.5 (range 8–45) and its correlation with size of the lithiasis was low (0.25). Postoperative value of COSQ was significantly lower (*p* < 0.001), with a mean value of 4.5 (range 0–16).

The preoperative COSQ values and their postoperative improvement (COSQ pre vs. COSQ post) showed a good correlation between each patient. The variation in COSQ value was not influenced by gender or the size of the stone. On the contrary, the ratio of the size of the residual hilar dilatation showed a negative correlation ( – 0.207), so that the smaller the hilar dilatation post-surgery, the greater the decrease in COSQ values.

## Discussion

Many factors contribute to the higher prevalence of sialoliths in the hilum of the submandibular gland, including an intervening mylohyoid muscle that sharply bends the duct from its cervical portion to its intraoral portion, and an anti-gravitational salivary flow. Bilateral SHL may occur (estimated 3%)[4], a fact that does not happen in our series.

Our findings support the importance of SHL patients’ quality of life and its postoperative improvement with transoral intervention. As a result, the average of preoperative COSQ value was 22.5. This was significantly reversed after surgery, with mean COSQ values of 4.5. Meaning that, once the obstruction is resolved, its condition becomes almost asymptomatic. The improvement in the QoL of patients undergoing TOSL has not been previously specifically described, as far as we know [9, 11, 18]. COSQ is a psychometric validated specific questionnaire for the study of the quality of life of patients with COS [17], likewise in the case of SHL. It can therefore be confirmed that TOSL solves the obstructive problem and significantly improves the QoL in all patients. As a result, it confirms that the transoral approach to LHS is an ideal technique for improving the quality of life of COS patients. Given these findings, glandular excision should not be the first option for treating COS caused by SHL.

The theoretical technical complexity is one of the reasons given for not carrying out a transoral technique. Despite in some moments of the procedure we work on a small, lax, and too easily movable surgical field, the anatomical complexity in the hilum of the submandibular gland is low. Although the stone itself frequently serves as the path of dissection because it is large and easily palpable, previous location of the lingual nerve is necessary to reduce the risk of injury. Furthermore, the nerve's previous identification permits the excision of the posterior portion of the sublingual gland at that level. Following that, the control of the surgical field and the localization of Wharton's duct are notably easier.

Compared to submandibular gland resection, transoral approach complications are both less frequent and severe [7, 9]. The complications reported by other authors are mainly related to the limited access rather than to the anatomical complexities of the dissection, which can lead to poor control of the lingual nerve isolation [5]. In our series, no patients suffered deep cervical or base-of-tongue complications, nor impact in lingual sensitivity. Additionally, transoral surgery is usually carried out in outpatient basis, which lowers the procedure's cost. The postoperative period is more bearable for the patient with transoral approaches, and they can return to their regular activities faster since the postoperative pain is tolerable.

Only few series study the possibility of persistence of lithiasis, always by ultrasound [9, 10, 19], reaching 11.3–16% [9, 19], similar to our series. In such cases, revision sialendoscopy may be performed to remove residual lithiasis or dilate a potential stricture, which was successfully performed in four of our patients. Therefore, to avoid this persistence, it is recommended to perform a sialendoscopy before the end of the transoral procedure to confirm the absence of stones distal to the TOSL incision [19]. In cases when the stone was fragmented during surgery or when several calculi were removed, there is a significant risk of persistent salivary calculus, according to Gerni et al*. *[19]. This idea should be expanded to include instances where the lithiasis is attached to a fibrotic ductal wall, as a result of several inflammatory episodes.

Other studies performed postoperative sialography, additionally to evaluate the integrity of the duct [6, 15]. According to Woo et al*. *[6] the majority of the ducts returned to its normal size by 3 months. In patients with stones with diameter smaller than 10 mm, sialography revealed a fully healed and intact duct. However, saccular duct pooling was revealed in patients with stones larger than 10 mm in diameter. Only one patient of Woo series presented persistent symptoms after surgery, confirming a postoperative hilar stenosis. As a result, similar to our findings, the presence of a saccule or a ductal cistern does not have to be associated with general symptoms of obstruction, and it should be regarded as an optimal functional result [6]. However, in our series, despite a remarkable improvement in postoperative COSQ values, the residual dilatation of the hilum has a negative influence on the reduction of COSQ values. Thus, patients with less postoperative hilar dilatation reduce their COSQ values much more. This decrease in the reduction of COSQ values may be due to the fact that the cistern causes an alteration in salivary flow; causing mild symptoms. Therefore, periodic self-massage and intraductal lavages may be helpful and may be recommended [20].

In the studies published to date, MR-Si has not previously been used as a postoperative control test. Given that knowledge about MR-Si is still becoming more widespread, it was imperative to study possible interobserver variability. According to our results, no significant differences were found between the two radiologists in terms of assessment of persistence of lithiasis, stone and ductal dilation measurement, duct recanalization or modification of parenchymal status after surgery. Therefore, MR-Si is a feasible and reliable control test in the pre- and post-surgical assessment of SHL, previously confirmed globally for COS [16].

It is noteworthy that all the patients of our series showed complete recanalization of the duct, independently of the state of hilar dilation. Therefore, it can be assured that TOSL allows the complete resolution of the obstructive problem, without generating a salivary fistula to the floor of the mouth. Similarly, given this complete recanalization, marsupialisation of the duct does not appear to be necessary; contrary to what has been proposed by other authors [11].

Besides, no specific studies on gland parenchyma have previously been performed beyond the presence of intraglandular duct dilatation, stone persistence, or intraparenchymal lithiasis. All of them were performed by ultrasound [9, 10], and never by MR-Si. Our study has proven that TOSL significantly improves the glandular parenchyma's condition, with marginal progression to parenchymal atrophy. Indeed Zhao et al. [8] used sialometry to measure gland function and found no differences between the affected gland and the contralateral gland control, and Makdissi et al. obtained similar results using scintigraphy [21]. Despite the postoperative sialographic findings, this demonstrated that the salivary gland function was adequate and comparable to the contralateral healthy gland.

## Conclusions

TOSL must be considered as the first treatment option for the management of SHL before removal of the submandibular gland, resulting in improvement of parenchymal inflammatory changes, recanalization of Wharton’s duct and enhancement of the patient's quality of life.

## References

[1] Huoh KC, Eisele DW (2011). Etiologic factors in sialolithiasis. Otolaryngol-Head Neck Surg Off J Am Acad Otolaryngol-Head Neck Surg.

[2] Sigismund PE, Zenk J, Koch M, Schapher M, Rudes M, Iro H (2015). Nearly 3000 salivary stones: some clinical and epidemiologic aspects. Laryngoscope.

[3] Sánchez Barrueco A, López-Acevedo Cornejo MV, Alcalá Rueda I, López Andrés S, González Galán F, Díaz Tapia G, et al (2022) Sialolithiasis: Mineralogical composition, crystalline structure, calculus site, and epidemiological features. Br J Oral Maxillofac Surg. S0266–4356(22)00239-X 10.1016/j.bjoms.2022.08.005 36109276

[4] Lustmann J, Regev E, Sialolithiasis MY (1990). A survey on 245 patients and a review of the literature. Int J Oral Maxillofac Surg.

[5] Saga-Gutierrez C, Chiesa-Estomba CM, Larruscain E, González-García JÁ, Sistiaga JA, Altuna X (2019). Transoral sialolitectomy as an alternative to submaxilectomy in the treatment of submaxillary sialolithiasis. Ear Nose Throat J.

[6] Woo SH, Kim JP, Kim JS, Jeong HS (2014). Anatomical recovery of the duct of the submandibular gland after transoral removal of a hilar stone without sialodochoplasty: evaluation of a phase II clinical trial. Br J Oral Maxillofac Surg.

[7] Combes J, Karavidas K, McGurk M (2009). Intraoral removal of proximal submandibular stones–an alternative to sialadenectomy?. Int J Oral Maxillofac Surg.

[8] Zhao YN, Zhang YQ, Zhang LQ, Xie XY, Liu DG, Yu GY (2020). Treatment strategy of hilar and intraglandular stones in Wharton’s duct: a 12-year experience. Laryngoscope.

[9] Capaccio P, Gaffuri M, Rossi V, Pignataro L (2017). Sialendoscope-assisted transoral removal of hilo-parenchymal sub-mandibular stones: surgical results and subjective scores. Acta Otorhinolaryngol Ital Organo Uff Della Soc Ital Otorinolaringol E Chir Cerv-facc.

[10] Shi H, Zhao J, Hze-Khoong EP, Liu S, Yin X, Hu Y (2020). A gland-sparing, intraoral sialolithotomy approach for hilar and intraparenchymal multiple stones in the submandibular gland. Sci Rep.

[11] Schapher M, Mantsopoulos K, Messbacher ME, Iro H, Koch M (2017). Transoral submandibulotomy for deep hilar submandibular gland sialolithiasis. Laryngoscope.

[12] Foletti JM, Graillon N, Avignon S, Guyot L, Chossegros C (2018). Salivary calculi removal by minimally invasive techniques: a decision tree based on the diameter of the calculi and their position in the excretory duct. J Oral Maxillofac Surg Off J Am Assoc Oral Maxillofac Surg.

[13] Eun YG, Chung DH, Kwon KH (2010). Advantages of intraoral removal over submandibular gland resection for proximal submandibular stones: a prospective randomized study. Laryngoscope.

[14] Galli P, Ceva A, Foletti JM, Iline N, Giorgi R, Chossegros C (2021). Salivary gland lithiasis recurrence after minimally-invasive surgery: incidence, risk factor and prevention. Laryngoscope.

[15] Liu DG, Jiang L, Xie XY, Zhang ZY, Zhang L, Yu GY (2013). Sialoendoscopy-assisted sialolithectomy for submandibular hilar calculi. J Oral Maxillofac Surg Off J Am Assoc Oral Maxillofac Surg.

[16] Sánchez Barrueco Á, SantillánCoello JM, González Galán F, Alcalá Rueda I, Aly SO, SobrinoGuijarro B (2022). Epidemiologic, radiologic, and sialendoscopic aspects in chronic obstructive sialadenitis. Eur Arch Oto-Rhino-Laryngol Off J Eur Fed Oto-Rhino-Laryngol Soc EUFOS Affil Ger Soc Oto-Rhino-Laryngol - Head Neck Surg.

[17] SantillánCoello JM, Sánchez Barrueco Á, González Galán F, Díaz Tapia G, Mahillo Fernández I, VillacampaAubá JM (2022). Validation of a Spanish chronic obstructive sialadenitis quality of life questionnaire (CSOC). Acta Otorrinolaringol Esp.

[18] Gillespie MB, O’Connell BP, Rawl JW, McLaughlin CW, Carroll WW, Nguyen SA (2015). Clinical and quality-of-life outcomes following gland-preserving surgery for chronic sialadenitis. Laryngoscope.

[19] Gerni M, Foletti JM, Collet C, Chossegros C (2017). Evaluation of the prevalence of residual sialolith fragments after transoral approach of Wharton’s duct. J Cranio-Maxillo-fac Surg Off Publ Eur Assoc Cranio-Maxillo-fac Surg.

[20] Harrison JD (2009). Causes, natural history, and incidence of salivary stones and obstructions. Otolaryngol Clin North Am..

[21] Makdissi J, Escudier MP, Brown JE, Osailan S, Drage N, McGurk M (2004). Glandular function after intraoral removal of salivary calculi from the hilum of the submandibular gland. Br J Oral Maxillofac Surg.

